# Flavokawain A Reduces Tumor-Initiating Properties and Stemness of Prostate Cancer

**DOI:** 10.3389/fonc.2022.943846

**Published:** 2022-07-13

**Authors:** Liankun Song, Merci Mino, Jana Yamak, Vyvyan Nguyen, Derron Lopez, Victor Pham, Ali Fazelpour, Vinh Le, Dongjun Fu, Matthew Tippin, Edward Uchio, Xiaolin Zi

**Affiliations:** ^1^ Department of Urology, University of California, Irvine, Orang, CA, United States; ^2^ Department of Pharmaceutical Sciences, University of California, Irvine, Irvine, CA, United States; ^3^ Chao Family Comprehensive Cancer Center, Orange, CA, United States

**Keywords:** Flavokawain A, Chemoprevention, prostate cancer, cancer stem cells, Neddylation

## Abstract

We have previously demonstrated the *in vivo* chemopreventive efficacy of flavokawain A (FKA), a novel chalcone from the kava plant, in prostate carcinogenesis models. However, the mechanisms of the anticarcinogenic effects of FKA remain largely unknown. We evaluated the effect of FKA on prostate tumor spheroid formation by prostate cancer stem cells, which were sorted out from CD44+/CD133+ prostate cancer cells 22Rv1 and DU145. FKA treatment significantly decreased both the size and numbers of the tumor spheroids over different generations of spheroid passages. In addition, the dietary feeding of FKA-formulated food to Nonobese diabetic/severe combined immunodeficiency (NOD/SCID) mice bearing CD44+/CD133+ 22Rv1 xenograft tumors resulted in a significant reduction of tumor growth compared to those fed with vehicle control food–fed mice. Furthermore, the expression of stem cell markers, such as Nanog, Oct4, and CD44, were markedly downregulated in both tumor spheroids and tumor tissues. We also observed that FKA inhibits Ubc12 neddylation, c-Myc, and keratin-8 expression in both CD44+/CD133+ prostate tumor spheroids and xenograft tumors. Our results suggest that FKA can reduce the tumor-initiating properties and stemness of prostate cancer, which provides a new mechanism for the chemoprevention efficacy of FKA.

## Introduction

Prostate cancer is the most diagnosed cancer in aging men and the second most common form of cancer-related death after lung and bronchus cancer in the United States. It has been estimated that 268,490 new cases of prostate cancer and 34,500 deaths occurred in the United States in 2022 (1). Most prostate cancers are curable with surgery or radiotherapy. However, approximately 20%–30% of patients with prostate cancer will experience disease recurrence or progress, but their survival rates are high; 5-, 10-, and 15-year-relative survival rates are nearly 100%, 98% and 95%, respectively (1). Because of the features of high prevalence, slow progression, and long latency in prostate cancer, dietary/lifestyle changes or no or less toxic chemoprevention approaches have also been considered to be critical for the prevention of human prostate cancer occurrence and progression (2).

Prostate cancer stem cells (CSCs) are a small subset of cells that have normal stem cell features of self-renewal and proliferative capacities and differentiation into heterogeneous lineages of cancer cells for the initiation and maintenance of prostatic neoplastic lesions (3). Increasing evidence indicates that CSCs contribute to the resistances of hormonal therapies, chemotherapy, chemoresistance, and radiotherapies, as well as to the recurrence and metastasis of cancer cells (3). Therefore, targeting CSCs and the eradication of the CSC pool would be an important strategy to treat or prevent cancer from its roots.

Flavokawain A (FKA) is a novel chalcone isolated from the roots of the kava plant that has been used for preparing kava beverages for thousands of years (4, 5). The consumption of kava beverages was linked to low cancer incidences, including prostate cancer, in South Pacific Island nations (5–7). Our previous study has demonstrated the chemoprevention efficacy of dietary FKA in the autochthonous transgenic adenocarcinoma of the mouse prostate model by the inhibition of the formation of high-grade prostatic intraepithelial neoplasia lesions and prostate adenocarcinomas and by the complete abolishment of distant organ metastasis (8). In addition, we have shown that FKA can act as a neddylation inhibitor to cause the degradation of S-phase kinase–associated protein 2 (Skp2) protein and the downregulation of androgen receptor (AR) expression (8). However, the effect of FKA on CSCs has not been reported yet.

In this study, we observed that FKA treatment decreased both the size and numbers of the tumor spheroids growing from CD44+/CD133+ tumor stem cells, which were sorted out by flow cytometry sorting (FACS) from the bulk cultures of prostate cancer DU145 and 22Rv1 cells. In addition, the growth of the 22Rv1CD44+/CD133+ xenograft tumors were significantly attenuated in FKA diet–fed mice compared to vehicle control diet–fed mice. The expression of the stem cell markers Nanog, Oct4, and Sox2 in tumor tissues and tumor spheroids were also markedly downregulated by FKA treatment.

## Materials and Methods

### Materials

Authenticated 22Rv1 and DU145 prostate cancer cell lines free of mycoplasma contamination were obtained from the American Type Culture Collection (ATCC) (Manassas, VA, USA) and used within 20 passages as previously described (9). FKA was isolated and purified by LKT Laboratories, Inc. (St. Paul, MN, USA) from the kava root extracts. MLN4924 was purchased from MedChemExpress LLC. (Monmouth Junction, NJ, USA). DMEM/F12, RPMI1640, penicillin–streptomycin, supplement B27 and N2, recombinant human fibroblast growth factor-basic (rhFGF-b), recombinant human epithelial growth factor (rhEGF), accutase, and fetal bovine serum (FBS) were purchased from Fisher Scientific (Hampton, NH, USA). The primary antibodies against Nanog and Keratin 8 (CK8) were obtained from Cell Signaling Technology (Danvers, MA, USA), anti-Sox2 from Life Technology Corporation (Carlsbad, CA, USA), anti-Oct4 from Abcam (Waltham, MA, USA), and anti-β-tubulin from Santa Cruz Biotechnology Inc. (Dallas, TX, USA), respectively.

#### Flow Cytometry Sorting and Prostasphere Formation Assays

Five to ten million of 22Rv1 and DU145 cells were stained with phycoerythrin (PE)- conjugated anti-CD44 and allophycocyanin-conjugated anti-CD133 antibodies (BioLegend, San Diego, CA, USA), and then, double-stained cells were sorted as the CSC population using an FACS machine (Laguna Hills, Becton-Dickson, CA, USA). A CSC single-cell suspension was split into a low-attachment 6-well plate at 500 cells per well, cultured in serum-free DMEM/F12 media supplemented with 2% B27, 1% N2, 10-ng/ml rhFGF-b, and 20-ng/ml rhEGF, and then treated with 0.01% Dimethyl sulfoxide (DMSO) (vehicle control) or FKA or MLN4924 at indicated concentrations once for 24 h or multiple times by refreshing the treatments every 3 days. After 14 days, spheres were digested with accutase and replated into new plates for culturing for another 14 days without any treatments as the secondary generation; the prostaspheres from the secondary generation were repeated with the same culturing process to evaluate the tertiary generation (10–12). The number and diameter of prostaspheres were counted and measured in five independent fields per well under a KEYENCE VHX-7000 microscope at different time points to evaluate the growth of prostaspheres.

#### Western Blot Analysis

DU145 and 22Rv1 bulk cultures and CSC prostaspheres at the secondary generation were treated with control, FKA, or MLN4924 at indicated concentrations for 48 h. Prostaspheres and prostate cancer cells were lysed, and tumor tissues homogenized in an Radioimmunoprecipitation Assay Buffer (RIPA) buffer with protease inhibitors and protein concentrations were determined by a Bio-Rad detergent compatible (DC) protein assay. An equal total amount of protein was loaded onto the SDS-PAGE gel and transferred onto a Polyvinylidene difluoride (PVDF) membrane. After blocking with 5% non-fat milk, the membranes are probed with indicated primary antibodies followed by appropriate secondary antibodies and by an enhanced chemiluminescence detection system for visualization. Western blotting analysis (9) was used to determine the protein expression levels of Nanog, Oct4, Sox2, c-Myc, CK8, Ubc12, β-tubulin, and Nedd8-Ubc12 in CSC prostaspheres and tumor tissues. The Western blotting bands were semi-quantified using Image J and adjusted for loading control, β-tubulin.

#### *In Vivo* Xenograft Tumorigenesis Assay

Ten thousand of sorted CD44+/CD133+ 22Rv1 cells in PBS were mixed with Matrigel from Corning Inc. (Corning, NY, USA) and injected subcutaneously into the right flank of NOD/SCID mice from Charles River Laboratories (Wilmington, MA, USA). Then, the mice were fed with a control diet or special diet supplemented with FKA (0.6%, w/w) as described previously (8). A University of California, Irvine–approved protocol (#2007-2740) was followed for animal care and treatments. The body weights and tumor volumes were measured every 3 days and food consumption recorded weekly as described in our previous publications (4). The mice were sacrificed when reaching a volume of 2,000 mm^3^ and tumor and organ weights recorded.

#### Immunohistochemistry and Immunofluorescence Staining Assays

Tumor tissues are fixed in 10% formalin, paraffin-embedded, and sectioned at 5-μm thickness. Section are deparaffinized, hydrated, and incubated in a steamer for 20 min in a sodium citrate buffer for antigen retrieval. Then, they are quenched with 3% hydrogen peroxide for 10 min followed by washing thrice using PBS and incubated with 3% normal goat serum blocking for 1 h. After that, the slides were incubated with primary antibodies, including Ki67 (1∶100), Nanog (1:150), CK8 (1:200), and OCT4 (1:200) overnight at 4°C. After rinsing with 0.1% tween 20-PBS for three times and incubating with an horseradish peroxidase (HRP)-labeled secondary antibody, sections were visualized with 3, 3-diaminobenzidine using the Cell and Tissue Staining kit (R&D Systems, Minneapolis, MN) and cover-slipped (8).

Fluorescence immunostaining was performed with an anti-CD44-PE antibody as described previously (13) without quenching in the dark, and the slides were counterstained with 4′,6-diamidino-2-phenylindole. All images were taken under a Keyence VHX-7000 microscope and quantified with Image J software.

Images were taken under both ×4 and ×40 objective lens of the Keyence VHX-7000 microscope. The numbers of positive-stained cells per field under ×40 objective lens were counted and averaged from 25 fields of five tumors in each group. The percentages of the control were calculated by dividing the average numbers of positive cells in the FKA-treated group by those in the control group. Error bars were standard deviations.

#### Statistical Analysis

Data were presented as means ± standard deviation or standard errors. The comparisons of prostasphere size and number between FKA and control treatments were performed using Student’s t-test. Repeated-measure analysis of variance was used to examine the differences in tumor sizes among treatments, time points, and treatment–time interactions. Additional post-tests were done to examine the differences in tumor sizes between control and FKA treatment at each time point by using the conservative Bonferroni method. All statistical tests were two sided.

## Results

### Flavokawain A Inhibits Prostasphere Formation by CD44^+^/CD133^+^-Positive Prostate Cancer Stem Cells and Bulk Prostate Cancer Cells

**Figure 1:**
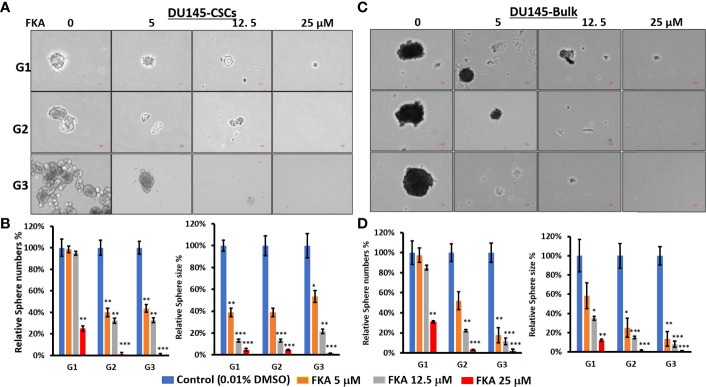
Flavokawain A (FKA) inhibits prostasphere formation. **(A)** Spheres generated from DU145 cancer stem cells (CSCs) with indicated treatment every 3 days for 14 days at G1 generation and without any treatments at G2 and G3 generations were imaged, and the relative sphere numbers and sizes were analyzed as shown in **(B, C)** Spheres generated from DU145 bulk cells with indicated treatments every 3 days for 14 days at G1 generation and without any treatments at G2 and G3 generations were imaged, and the relative sphere numbers and sizes were analyzed as shown in **(D)**. Scale bar: 50 µm. *P<0.5, **P<0.01, ***P<0.001.

CSCs accounting for a very small portion (0.01%–2%) of the total tumor cells have been identified by different surface markers (10, 14, 15). CD133 and CD44 cell surface markers are putative CSC markers for different tumors, including prostate cancer. The coexpression of CD44 and CD133 has been shown to be related to worse clinical outcomes and the survival of cancer patients, including those diagnosed with prostate cancer (10, 14, 15). Using FACS sorting, approximately 0.05% and 0.02% of CD133+/CD44+-positive CSCs from the bulk culture of DU145 and 22Rv1 cells were obtained (**Supplementary Figure 1**). These CSCs in parallel with unsorted bulk cells were cultured on low-attachment 6-well plates to generate prostaspheres for three generations. **Figures 1A, C** show that there are significant alterations in morphology between the primary generation and secondary or tertiary generation of the CSC-generated prostaspheres, whereas there are no significant changes on the morphology of bulk cell–generated tumor spheres. The CSC-generated prostaspheres exhibit mixed branching and mass phenotype structures in their secondary and tertiary generations, which suggest self-renewal and differentiation.

In addition, FKA treatment resulted in a dose-dependent reduction of both the sizes and numbers of the prostaspheres in DU145 and 22Rv1 CSCs (**Figures 1B, D** and **Supplementary Figures 2B, D**). Both multiple doses (every 3 days, **Figure 1** and **Supplementary Figure 2**) and single doses (only once at the beginning of the prostasphere formation assay, **Supplementary Figure 3**) of FKA treatment at their primary generation similarly decreased the sizes and numbers of the prostaspheres. The inhibitory effects of FKA on the prostaspheres were maintained on the secondary and tertiary generations of the CSCs when no further FKA was added (**Figure 1** and **Supplementary Figures 2, 3**). FKA is also more effective in reducing the size of the tumor spheroids compared to its effect on the numbers. FKA treatment at a 5-μM concentration almost completely inhibited the branching of the DU145 and 22Rv1 CSCs at their tertiary generation (**Figure 1** and **Supplementary Figure 2**). The observed branching morphology at the secondary and tertiary generations of DU145 and 22Rv1 CSC prostaspheres may suggest that these CSCs undergo differentiation. Taken together, these results suggest that FKA may inhibit the capacity of the renewal and differentiation of DU145 and 22Rv1 CSCs.

### Flavokawain A Inhibits the Expression of Putative Cancer Stem Cell Markers

**Figure 2:**
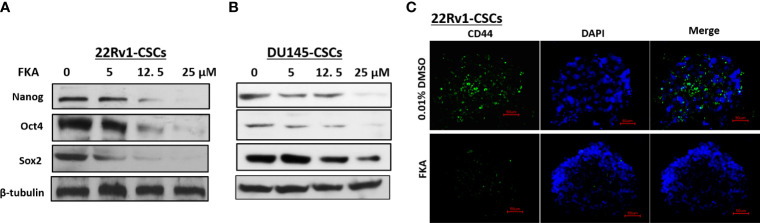
Effects of FKA on the expression of stem cell markers in prostaspheres. **(A, B)** FKA treatment of prostaspheres generated from DU145 and 22Rv1 CSCs at the secondary generation at indicated concentrations for 24 h decreased the expression of Nanog, Oct4, and Sox2. **(C)** FKA treatment at 12 µM for 24 h significantly reduced the number of CD44- positive cells in prostaspheres generated from 22Rv1 CSCs.

Oct4, Nanog, and Sox2 are highly expressed in CSCs in different cancers and recognized as the most important transcriptional factors to regulate stem cell pluripotency, renewal, and maintenance (16, 17). Western blotting analysis reveals that the FKA treatment of prostaspheres generated from DU145 and 22Rv1 CSCs for 24 h resulted in a dose-dependent decrease in the protein expression of Oct4, Sox2, and Nanog (**Figures 2A, B**). The expression of Oct4 and Nanog was significantly downregulated by FKA at a concentration of 5 µM, and Sox2 expression decreased markedly at the FKA concentrations of 12.5 and 25 µM (**Figures 2A, B**). In addition, the immunofluorescence density of CD44, a cell surface maker of CSCs, also decreased dramatically after FKA treatment (**Figures 2C**). These results further confirm that FKA suppresses the stemness of prostate CSCs in cell cultures.

### Dietary Feeding of Flavokawain A Suppresses Prostate Cancer Stem Cell–Initiated Tumorigenesis in a Xenograft Model

**Figure 3:**
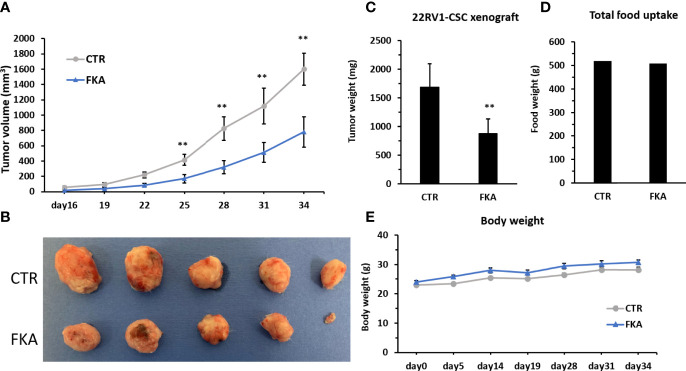
Dietary FKA inhibit prostate CSC–initiated tumor growth. **(A)** Mice were each subcutaneously injected with ten thousand of sorted CD133 and CD44 double-positive 22Rv cells and randomized into the vehicle control (CTR) diet and 0.6% FKA-formulated diet. Tumor volumes were measured every 3 days and means ± standard errors (SEM) are shown. **(B)** Photographs of each tumor at the end of treatment. **(C)** Tumor weights from both groups were measured and analyzed at the end of the treatments. **(D)** Food uptake was measured every 3 days, and the total weights of food are shown in CTR and FKA groups. **(E)** Body weights of mice were recorded over time, and the mean body weights of each group are shown. Error bars represent SEMs (n = 5). **: P<0.01.

We were able to generate rapid *in vivo* tumor growth in NOD/SCID mice with only ten thousand of CD133+/CD44+-positive CSCs from the bulk cultures of 22Rv1 cells, whereas ten million of 22Rv1 cells from unsorted bulk cultures are generally required to successfully generate rapid tumor growth in the same strain of mice. The CD133+/CD44+-positive 22Rv1 CSCs are approximately 100 times more potent in tumorigenicity compared to bulk 22Rv1 cells. NOD/SCID mice were each subcutaneously injected with ten thousand of CD133+/CD44+- positive 22Rv1 cells and then randomized into the vehicle control diet and 0.6% FKA-formulated diet groups. **Figure 3A** shows a progressive tumor xenograft growth in the vehicle control group, whereas the dietary FKA group exhibited a slower tumor growth rate. The wet tumor weights in the control and FKA-formulated diet groups recorded at the end of the treatment are 1,683.66 ± 368.84 g and 875.22 ± 226.68 g, respectively (mean ± SEM; n = 5 for control and n = 5 for FKA diet groups; P<0.01, Student’s t-test). The FKA-formulated diet reduced tumor growth by 48% at the end of the treatment (**Figures 3B, C**). There are no significant differences in body weight gains and food uptakes between mice fed with a control diet and FKA-formulated diet (**Figures 3D, E**).

### Dietary Feeding of Flavokawain A Reduces Proliferation and Stemness of Prostate Cancer Stem Cell–Initiated Tumorigenesis

**Figure 4:**
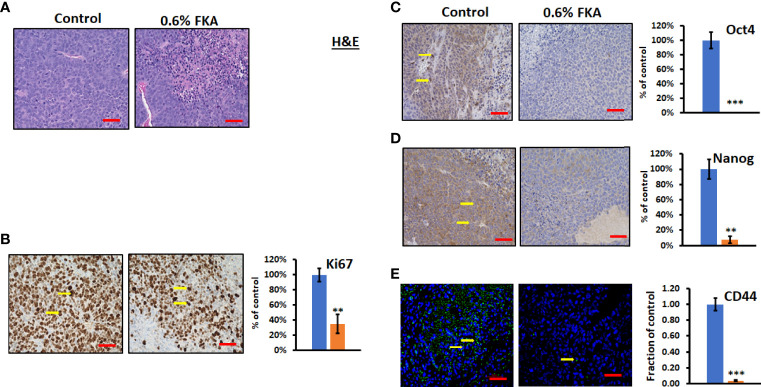
FKA inhibits the expression of CSC markers in CSC xenograft tumors. **(A)** H&E-stained tumor tissues. **(B, C)** IHC- or immunofluorescence-stained tissue sections by anti-Ki67, -Oct4, -Nanog, and -CD44 antibodies, respectively, were photographed at ×40 (scale bar 50 µm) magnifications, and the quantification of the positive staining cells (yellow arrows) of FKA-treated tumors relative to control-treated tumors was shown **P<0.01 and ***P<0.001.

H&E staining shows that CSC xenograft tumors in the control diet group exhibit a high density of proliferative cells, whereas there are many necrotic areas in the tumors from FKA diet–fed groups (**Figure 4A**). Immunohistochemical analysis reveals that sections from the dietary FKA–treated prostate cancer CSC xenograft tumors exhibited a significant reduction in the number of Ki67-positive cells by more than 64% compared to those from the vehicle control diet group (**Figure 4B**, **: P < 0.01). Notably, there are a highly significant to complete inhibition in numbers of Oct4 and Nanog-positive cells by 100% and 92%, respectively, in dietary FKA–treated tumors compared to vehicle control dietary treatment (**Figures 4C, D**, **: P< 0.01 or ***: P<0.001). In addition, a remarkable reduction in the number of CD44- positive cells by dietary FKA treatment by 96% was noticed compared to vehicle control treatment (**Figure 4E**, P<0.01). Tumor sections from FKA diet–fed mice also have less differentiation marker CK8 staining compared to those from control diet–fed mice (**Supplementary Figures 4C, D**). These results suggest that dietary FKA is highly effective in the inhibition of proliferation, differentiation, and cancer stemness in the tumors of the 22Rv1 CSC xenograft model.

### Neddylation Inhibitor, MLN4924, Suppresses Prostasphere Formation by CD44^+^/CD133^+–^Positive Prostate Cancer Stem Cells

**Figure 5:**
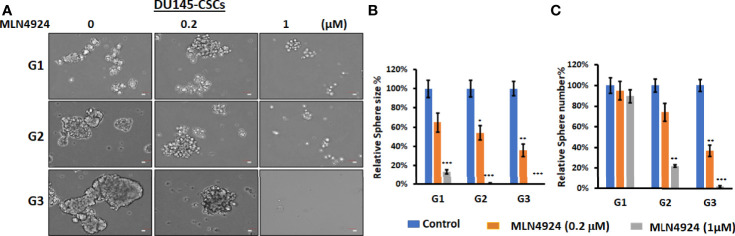
MLN4924 inhibits CSC prostasphere formation. **(A)** Prostaspheres generated from DU145 with indicated treatment every 3 days for 14 days at G1 generation and without any treatments at G2 and G3 generations were imaged, Scale bar: 50 µm. *P<0.5, **P<0.01, ***P<0.001. **(B, C)** The numbers and sizes of prostaspheres were analyzed, and percentage changes from FKA treatments relative to control treatments are shown.

We have previously shown that FKA is a novel neddylation inhibitor that inhibited NEDD8 conjugations to both Cullin1 and Ubc12 and Ubc12 neddylation in an *in vitro* assay (8). Other studies reported that MLN4924, the first-in-class inhibitor of the NEDD8-activating enzyme (NAE), suppressed the CSC properties in uveal melanoma, leukemia stem cells, and patient-derived glioblastoma stem cells (18–20). However, the effect of neddylation inhibition on prostate CSCs has not been reported. **Figure 5** and **Supplementary Figure 5** show that MLN4924 at concentrations of 0.2 and 1 μM significantly inhibited the sizes and numbers of prostaspheres and these inhibitory effects last for the secondary (G2) and tertiary (G3) generation of 22Rv1 and DU145 CSCs even without further addition of the inhibitor. These results further support that the inhibition of neddylation represses the self-renewal and differentiation of prostate CSCs.

### Flavokawain A Inhibits Neddylation and c-Myc and CK8 Expression in Prostate Cancer Stem Cells Both *In Vitro* and *In Vivo*

**Figure 6:**
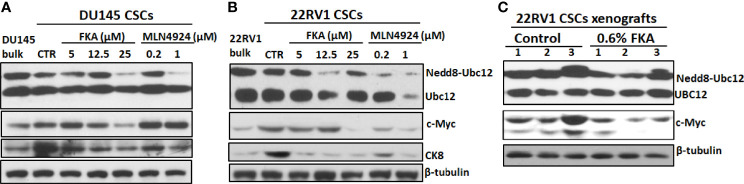
Inhibition of neddylation by FKA and MLN4924 downregulates the expression of c-Myc and CK8. **(A, B)** Representative pictures of the Western blotting analysis of Ubc12 neddylation and expression of c-Myc and CK8 in DU145 and 22Rv1 CSC prostaspheres at G2 after indicated concentrations of control, FKA and MLN4924 treatments for 48 h. **(C)** Western blotting analysis of Ubc12 neddylation and c-Myc expression in 22RV1 xenograft tumors from mice fed with control and FKA formulated diet for 18 days. Experiments were repeated thrice, and β-tubulin was used as a loading control.

Zhou et al. (21) reported that MLN4924 at concentrations of 0.1 and 0.3 μM induced c-Myc expression in H125, MCF7, and H1299 cells in two-dimensional (2D) cultures and c-Myc has been shown to play an important role in prostate CSCs (22). **Figures 6A, B** show that DU145 and 22Rv1 CSCs have enhanced levels of c-Myc protein in prostaspheres compared to their bulk cultures in 2D. FKA treatments decreased the protein expression of c-Myc in a dose-dependent manner in prostaspheres generated from both DU145 and 22Rv1 CSCs (**Figures 6A, B**). However, MLN4924 at 0.2- and 1-μM concentrations has no effect on the protein expression of c-Myc in DU145 prostaspheres but decreased the expression of c-Myc in 22Rv1 prostaspheres (**Figures 6A, B**), which suggest a context-dependent effect of MLN4924. FKA and MLN4924 also decreased the expression of the differentiation marker CK8 in both DU145 and 22Rv1 CSC prostaspheres (**Figures 6A, B**). This result is consistent with the reduced branching morphology by FKA and MLN4924 treatments as shown in **Figures 1A, C** and **Figure 5A** and **Supplementary Figure 5A**, suggesting that the inhibition of neddylation by FKA and MLN4924 may prevent CSC differentiation.

**Figure 6C** further shows that dietary FKA inhibited Ubc12 neddylation and the protein expression of c-Myc expression *in vivo* in the xenograft tumor tissues of 22Rv1 CSCs. These results supported the consistency of the *in vitro* effects of FKA in prostaspheres with the *in vivo* findings in xenograft tumors.

## Discussion

CSCs presenting in the bulk of tumors in small numbers typically under a quiescent status are extremely tumorigenic and have the properties of self-renewal and differentiation into different cell populations (3, 14, 16). Therefore, CSCs have been considered as a driver for cancer initiation, progression, metastasis, drug resistance, and recurrence (3, 14, 16). We have demonstrated that FKA, a novel chalcone from the kava plant, inhibits the growth of bulk tumor cells in different transgenic models and xenograft tumor models (6–8, 23). The bulk of tumors consists of heterogenous cell populations, including non-CSCs and CSCs. However, the effect of FKA on CSCs has not been reported yet. In this study, we are the first to report that FKA inhibits both the *in vitro* growth of prostaspheres and the *in vivo* growth of xenograft tumors generated from highly tumorigenic CSCs. Conventional therapies, such as chemotherapies and radiotherapy, often kills most non-CSCs, and then, small numbers of CSCs can survive through these treatments, leading to cancer progression, recurrence, and metastasis (3, 14, 16). Therefore, agents, like FKA, which target CSCs, may be useful for developing combined therapeutic approaches with conventional therapies.

Our previous study has demonstrated that FKA inhibited NEDD8 conjugations to both Cullin1 and Ubc12, delayed tumor progression, and eliminated distant metastasis in the TRMAP transgenic mouse model, although it was unclear whether the underlying mechanism was related to the inhibition of CSCs (8). Recently, Jin et al. (18) also reported that neddylation inhibition by MLN4924 completely abolished the liver metastasis of uveal melanoma through suppressing the CSCs properties of uveal melanoma and the inhibition of tumor angiogenesis. It was hypothesized that neddylation inhibition may block the establishment of clinically significant metastases through the eradication of CSCs (18, 24). Liu et al. (19) have shown that MLN4924 inhibited chronic myeloid leukemia by targeting leukemia stem cells. MLN4924 was also shown to effectively inhibit the proliferation of patient-derived glioblastoma stem cells with minimal toxicities to normal human astrocytes (20). However, the responses of these patient-derived glioblastoma stem cells to MLN4924 treatment are heterogenous (20). Yin et al. have identified that neddylation inhibition attenuated stem cell properties by inhibiting F-box/WD repeat-containing protein 2 (FBXW2), which resulted in Msh Homeobox 2 accumulation to repress stem cell marker SOX2 expression (25). In this study, **Figure 4** shows that dietary FKA resulted in a highly potent-to-complete inhibition of the expression of stem cell markers, such as CD44, Oct4, and Nanog, in 22Rv1 CSC-initiated tumors in NOD/SCID mice. Nevertheless, there are still many knowledge gaps to fully understand the mechanisms by which FKA regulates CSC markers and to elucidate the relationship between the effect of FKA on CSCs and prostate cancer metastasis. Therefore, further investigation into the relationship of neddylation inhibition by FKA, prostate CSCs, and prostate cancer metastasis is warranted.

The NEDD8-conjugating enzyme Ubc12 is another critical target along the neddylation pathway in addition to NAE for developing new therapies for the treatment of cancer. Importantly, Li et al. (26) has shown that Ubc12 knockdown by shRNAs effectively reduced the growth of MLN4924-resistant lung cancer cells. Several approaches, including Ubc12 knockdown, the overexpression of dominant-negative Ubc12 and chemical inhibitors of the Ubc12-DCN1 interaction, have been used to understand the cellular functions of Ubc12 in cancer cells and the selective effects on cullin–RING ligase substrates (26–30). The inhibition of Ubc12 by these approaches has been linked to G2 arrest in cell cycle progression, apoptosis, and autophagy. However, there is very little known about a role of the specific components of the neddylation pathway, such as Ubc12 neddylation, in CSCs. We have shown here that FKA and MLN4924 inhibited Ubc12 neddylation in both CSC prostaspheres and CSC-initiated tumors, accompanied by the loss of branching morphology and decreased expression of a differentiation marker CK8. These results suggest that the inhibition of Ubc12 neddylation by FKA and MLN4924 may be able to prevent CSC renewal and differentiation for keeping the dormancy of prostate cancer.

MYC is a family of transcription factors and proto-oncogenes, which plays a key role in prostate cancer initiation, early progression, and metastasis (22). MYC is also a key transcriptional factor for driving the induced pluripotent stem cell formation and self-renewal of embryonic stem cells (31). MYC has been reported to stimulate an embryonic stem cell–like expression signature in MYC-driven mouse prostate tumors (31). In addition, MYC is important in prostate cancer disparity in African-American (AA) men who are more frequently diagnosed with prostate cancer and have an aggressive disease. A risk locus at chromosome 8q24 mapping MYC oncogene is significantly associated with prostate cancer cases with a West African ancestry (32–34). MYC amplification is also more frequent in metastatic African American (AA) prostate cancer patients (35). **Figures 6A, B** show that 22Rv1 and DU145 CSCs express higher levels of c-Myc protein in their prostaspheres compared to their bulk cultures. FKA treatment reduced the expression of c-Myc protein both in prostaspheres and in 22Rv1 CSC–initiated xenograft tumors (**Figures 6A–C**). FKA, which is more effective to target Ubc12 neddylation, appears to act through a different mechanism for the inhibition of neddylation from MLN4924. In contrast to the inhibitory effect of FKA on c-Myc expression and the expression of stem cell markers, Zhou et al. (21) reported that MLN4924, an NAE inhibitor for neddylation inhibition, stimulated self-renewal, and promoted stem cell differentiation and teratoma formation *in vivo* in mice at its low concentrations (0.1–0.3 μM). In addition, MLN4924 increased the expression of stem cell markers, CD44 and Oct4, and caused c-Myc accumulation by blocking their degradation in an FBXW7-dependent manner. In this study, we have shown that MLN4924 at a similar concentration has no effect on or decreased the expression of c-Myc protein in prostaspheres. In these results along with other reports (18–20, 25), it appears that the effect of MLN4924 on CSCs and the expression of stem cell markers and c-Myc protein are context dependent and there exist significant differences in the biological and molecular mechanisms between FKA and MLN4924 as neddylation inhibitors to regulate CSCs. Further studies are thus needed to identify the effect of different components along the neddylation pathway on the regulation of CSC properties.

## Conclusions

In summary, we have demonstrated that FKA inhibits prostaspheres formation that were established from prostate CSCs and from bulk cultures of prostate cancer cells. Dietary FKA significantly reduced the *in vivo* growth of xenograft tumors generated from highly tumorigenic CSCs. Notably, FKA treatment resulted in a highly efficient-to-complete inhibition of the expression of stem cell markers, such as Nanog, Oct4, and CD44, in both prostaspheres and tumor tissues. FKA also inhibited the neddylation of Ubc12 and the expression of c-Myc, a key target for CSCs, and a differentiation marker CK8 in prostaspheres and tumor tissues. Our results suggest that FKA has the potential to target both differentiated cells and CSCs in the tumor mass, which would provide a better option to treat or prevent prostate cancer through both tumor mass reduction and the prevention of disease recurrence and metastasis. Further studies are still needed to investigate a role of neddylation inhibition by FKA in regulating c-Myc protein expression and the properties of prostate CSCs.

## Data Availability Statement

The raw data supporting the conclusions of this article will be made available by the authors, without undue reservation.

## Ethics Statement

The animal study was reviewed and approved by IACUC, University of California, Irvine.

## Author Contributions

Conception and design: XZ, EU. Development of methodology: LS, VP. Acquisition of data: LS, MM, JY, VN, DL, AF, VL, DF, MT. Analysis and interpretation of data: LS, XZ. Administrative, technical, or material support: XZ. Writing, review, and/or revision of the manuscript: LS, XZ. Study supervision: XZ. All authors contributed to the article and approved the submitted version.

## Funding

This work was supported in part by NIH award 5R01CA122558-05 and 5R01CA193967-05 (to XZ).

## Conflict of Interest

The authors declare that the research was conducted in the absence of any commercial or financial relationships that could be construed as a potential conflict of interest.

## Publisher’s Note

All claims expressed in this article are solely those of the authors and do not necessarily represent those of their affiliated organizations, or those of the publisher, the editors and the reviewers. Any product that may be evaluated in this article, or claim that may be made by its manufacturer, is not guaranteed or endorsed by the publisher.
