# Supplementary material for: Flavokawain A Reduces Tumor-Initiating Properties and Stemness of Prostate Cancer
Source: Front Oncol. 2022 Jul 13;12:943846. doi: 10.3389/fonc.2022.943846 (PMC9326116; doi:10.3389/fonc.2022.943846)
Supplement: Supplementary file 1 [file DataSheet_1.pdf]

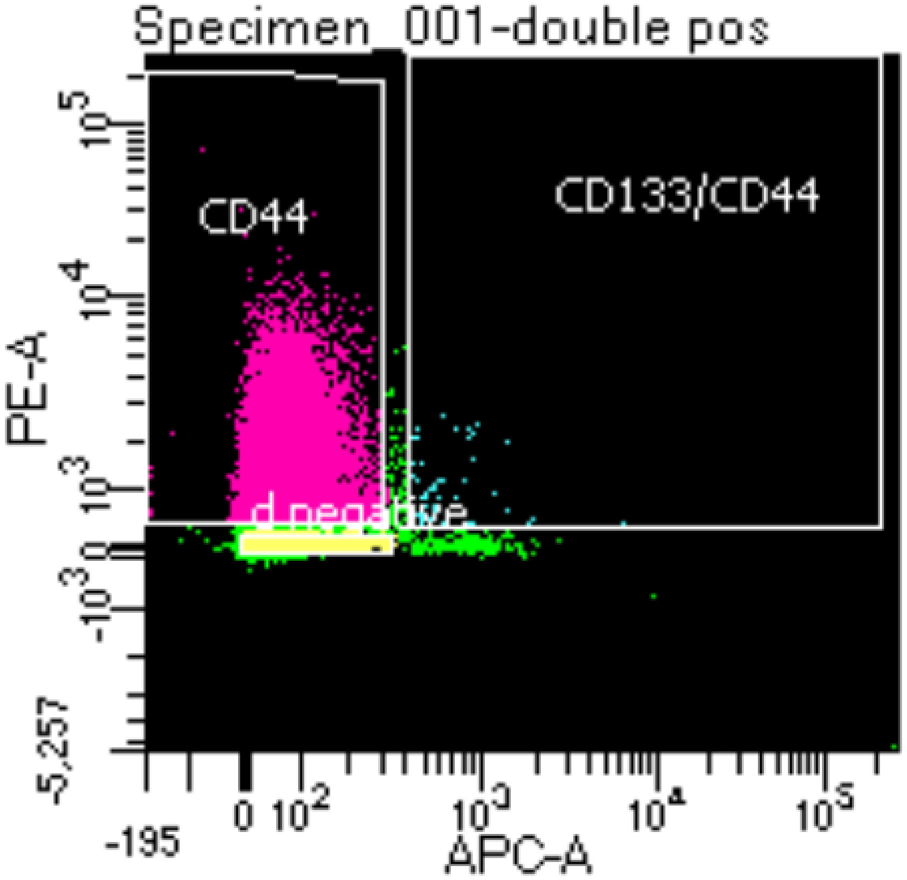

Supplementary Figure 1. Sorting gate for CSCs. DU145 and 22Rv1 cells were stained with PE conjugated anti-CD44 and APC conjugated anti-CD133 antibodies, CD44 and CD133 double positive cells were collected as CSCs.

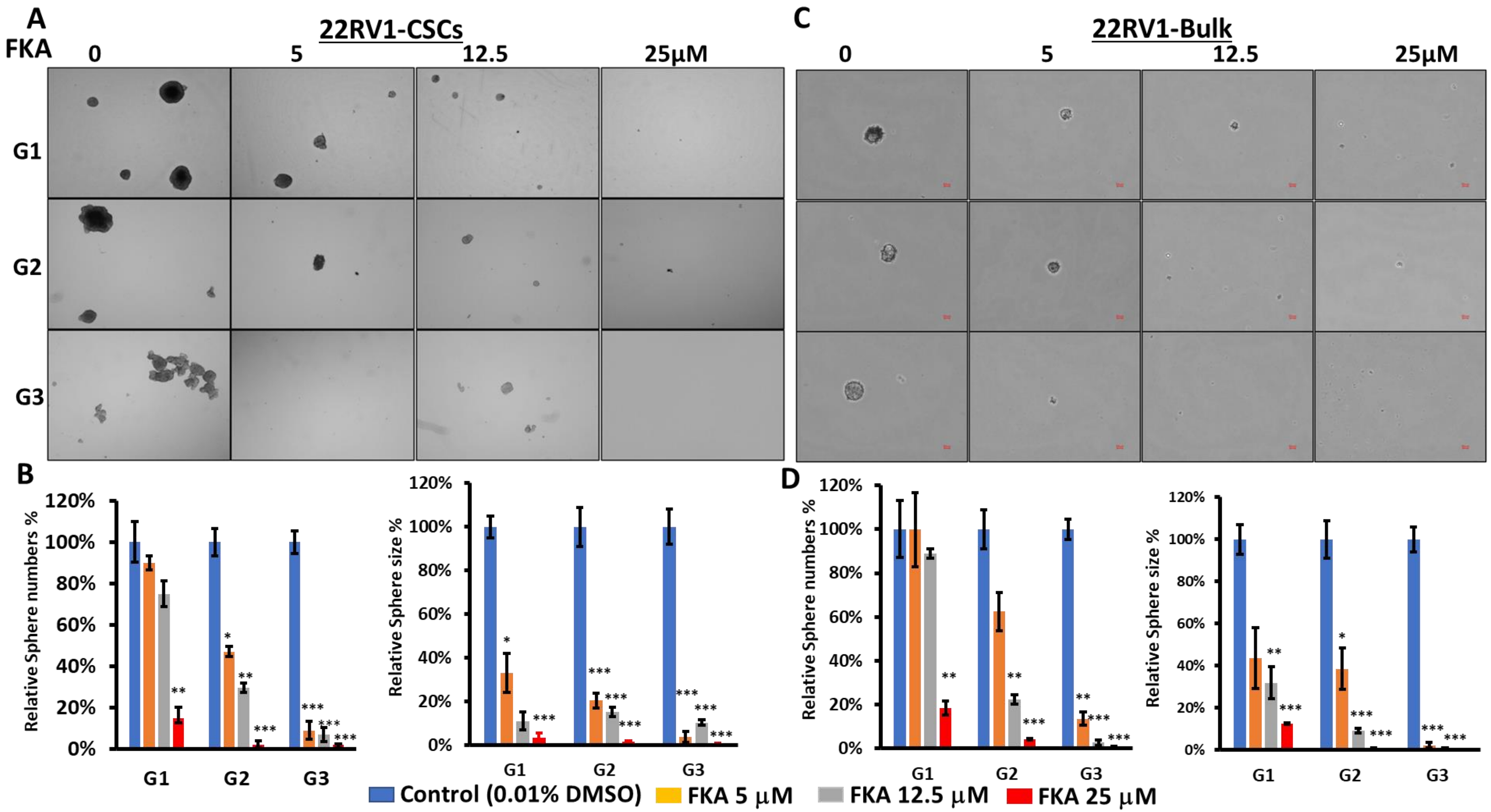

Supplementary Figure 2. FKA treatment at multiple dosing inhibit 22Rv1 prostaspheres formation. (A). The primary tumor spheres (G1) were generated from sorted CD44 and CD133 double positive 22Rv1 cells and treated with the indicated concentrations of FKA for 14 days with refreshing FKA every 3 days. Then the primary spheres from G1 were harvested, disassociated, and replated at the same cell density to generate G2 spheres for 14 days until G3. **(B).** Numbers and sizes of prostasphere sizes were analyzed during each passage. **(C, D).** 22Rv1 bulk cells were treated and analyzed similarly as A &B. Scale bar: 50µm. **P*<0.5, ***P*<0.01, ****P*<0.001.

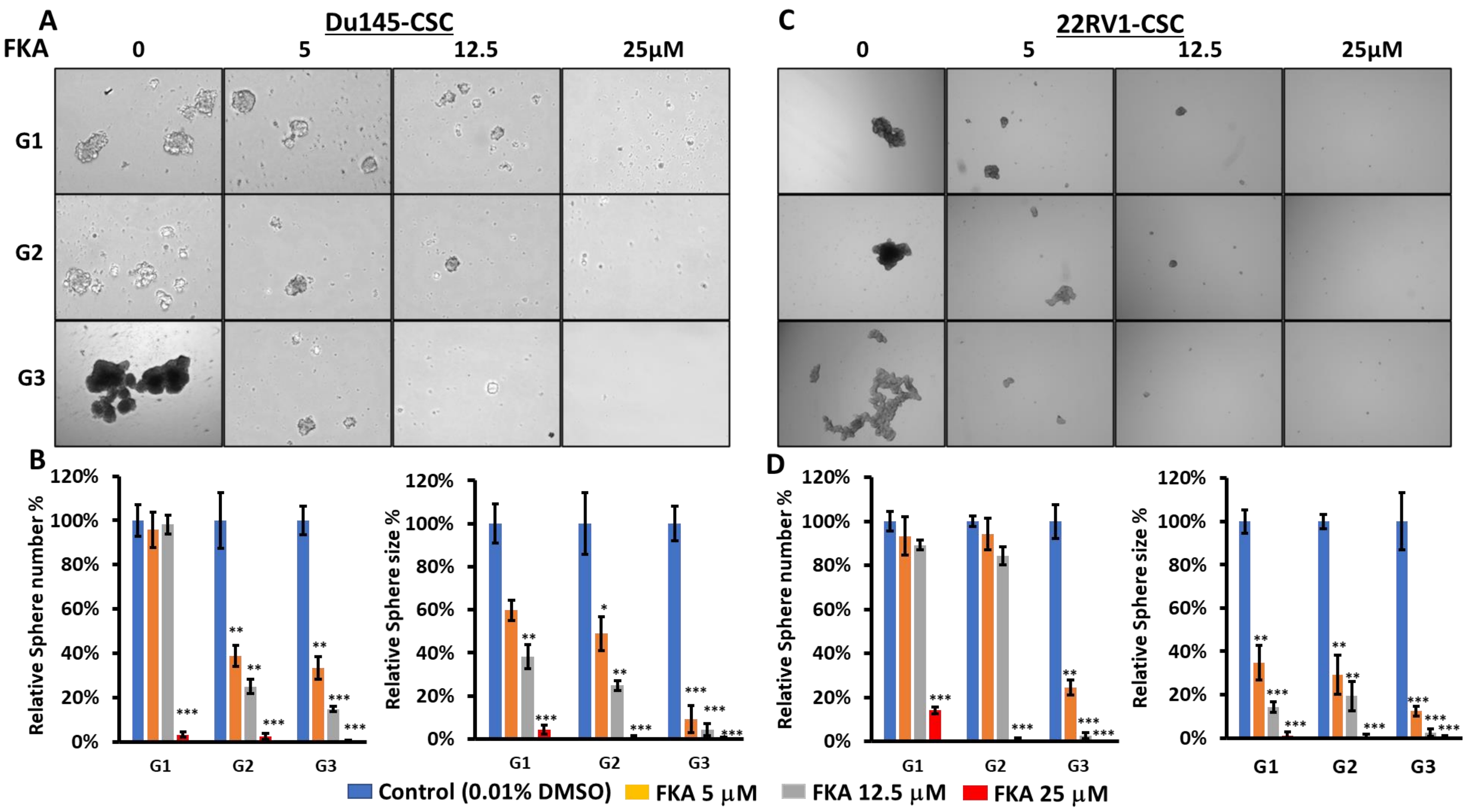

Supplementary Figure 3. FKA treatment at single dose suppresses prostaspheres growth. (A, C). The primary tumor spheres (G1) were generated and treated with the indicated concentration of FKA once and cultured for 14 days. Then the primary spheres from G1 were harvested, disassociated, and replated at the same cell density to generate secondary (G2) spheres for 14 d until tertiary (G3). Prostaspheres formation from Du145 CSCs **(B)** and 22Rv1 CSCs **(D)** was imaged every week, numbers and sizes of prostaspheres were analyzed during each passage. Scale bar: 50μm. **P*<0.5, ***P*<0.01, ****P*<0.001.

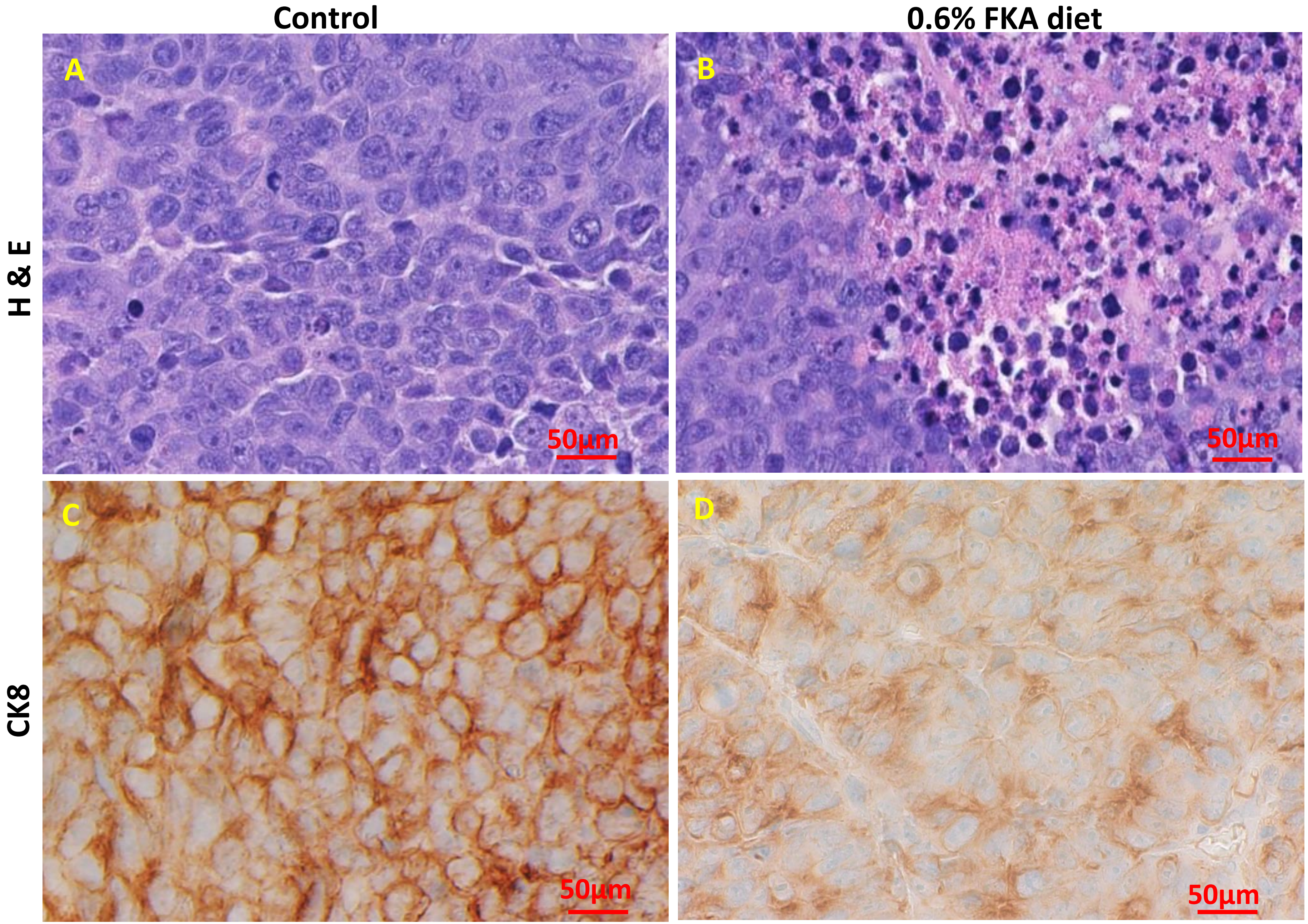

Supplementary Figure 4. FKA inhibits the expression of differentiation makrerCK8 in CSC xenograft tumors. (A, B). H&E-stained tumor tissues. (C, D) IHC stained CK8 in tumor sections from control or FKA diet fed mice.

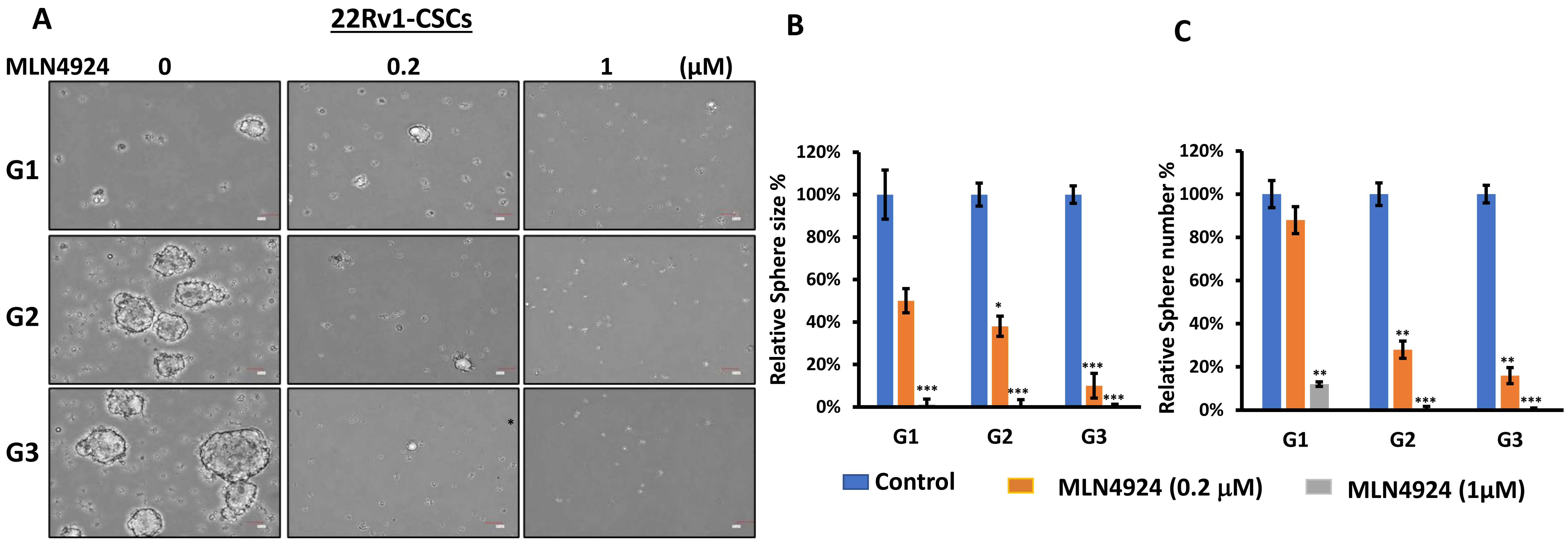

Supplementary Figure 5. MLN4924 inhibits CSCs prostaspheres formation. (A). Prostaspheres generated from 22Rv1 with indicated treatment every three days for 14 days at G1 generation and without any treatments at G2 and G3 generations were imaged, Scale bar: 50μm. *P<0.5, **P<0.01, ***P<0.001. (B&D). (B, C). The numbers and sizes of prostaspheres were analyzed and percentage changes from FKA treatments relative to control treatments are shown.
